# Beneficial role of gut microbes in maintenance of pace-of-life traits in *Phrynocephalus vlangalii*

**DOI:** 10.3389/frmbi.2022.962761

**Published:** 2022-11-21

**Authors:** Zhaohui Bing, Chenkai Niu, Cui Yang, Yue Qi, Yangyang Zhao, Shuhui Cao, Wei Zhao

**Affiliations:** School of Life Sciences, Lanzhou University, Lanzhou, China

**Keywords:** *Phrynocephalus vlangalii*, high altitude, behavior, lizard, fast pace, slow pace, pace-of-life, gut microbe

## Abstract

The pace-of-life syndrome theory suggests that species, populations, and individuals are positioned along a slow–fast pace-of-life continuum. However, whether and how individuals maintain a fast pace of life in a slow pace of life population remains unknown. In this study, the boldness and foraging behavior of *Phrynocephalus vlangalii* from Maduo (4250 m above sea level), a typical slow-paced population, were screened frequently. Both behaviors of *P*. *vlangalii* were significantly recurrent and linked with one another. Based on boldness and foraging behavior, the lizards were divided into positive and shy groups, and their gut microbial diversity were studied using 16S rRNA gene sequencing. No significant difference in α diversity was observed; however, a significant difference existed in the β diversity of gut microbes between the two groups. Principal coordinate analysis indicated that the gut microbes in the two groups were distinct. Linear discriminant analysis effect size determined that the shy group contained a more significant proportion of *Rikenellaceae* and *Clostridia*. In contrast, the positive group had a higher proportion of *Verrucomicrobiota*, *Verrucomicrobiae*, and *Akkermansiaceae*. Kyoto encyclopedia of genes and genomes pathway analysis revealed that biodegradation and metabolism, including lipid metabolism and glycan biosynthesis, were higher in the positive group; on the contrary, nucleotide metabolism and enzyme families were significantly higher in the shy group. The results showed that positive lizards had more beneficial intestinal microflora for lipid and glucose metabolism to satisfy their high metabolic energy demand, whereas shy lizards had more beneficial intestinal microflora for maintaining an elevated fasting blood glucose, a long life span, and a more stable metabolism to sustain their slow pace of life. In this study, we validate a strong relationship between the individual’s pace-of-life traits and intestinal microbiota in *P. vlangalii*. Further, we demonstrate that gut microorganisms are essential in sustaining the energy-intensive personality traits at high altitudes.

## 1 Introduction

The pace-of-life syndromes (POLSs) predict the behavioral and physiological heterogeneity among interspecies or interpopulations due to variations in life history strategies caused by environmental changes (Ricklefs and Wikelski, 2002). According to POLS theory, ecological factors largely influence the phenotypes associated with the trade-off between current and future reproduction, resulting in a slow-to-fast continuum of physiological and life history traits (Dammhahn et al., 2018). As a typical extreme environment, a plateau characterized by low temperature, poor oxygen, and intense ultraviolet rays imposes severe energy stress on native species, particularly ectotherms because they must spend more energy and time regulating their body temperature (Wang et al., 2017; Vicenzi et al., 2019). To deal with such energy-intensive constraints, animals at high altitudes often prioritize energy for their growth, reproduction, immunity, and other life activities (Rohr, 1997; Park et al., 2019), indicating that the life history strategies of these organisms are consistent with their slow pace of life (POL) by avoiding expensive phenotypic traits.

The POLS theory, by incorporating behavioral traits into the framework, can better explain the intrapopulation variation of life history strategies (Réale et al., 2010). According to the extended framework, the slow end of the POL continuum can be characterized by delayed reproduction and risk-averse behaviors (Campos-Candela et al., 2019; Dammhahn et al., 2018; Réale et al., 2010; Mathot and Frankenhuis, 2018; Silk and Hodgson, 2021). According to the performance model, animal behavior and energy metabolism are strongly linked; consequently, positive personality traits such as boldness and high aggressiveness require more energy to maintain energy-intensive behavior than do shy personalities (Biro and Stamps, 2008; Careau and Garland, 2012); thus, fast-paced individuals require more energy than slow-paced individuals (Hille and Cooper, 2015; Qu et al., 2019). Evidently, the environment determines the benefits of high metabolic activities and shapes the trade-off strategy in organisms that choose to maintain their POL.

The energy absorption capacity of many animals is enhanced to overcome energy limitations at high altitudes. Many studies confirm that the digestive system of animals that inhabit high-altitude areas has undergone evolutionary changes, including changes in the length of the digestive tract, morphological characteristics of small intestinal villi, and the number of argyrophil cells (Tang et al., 2013; Murray, 2016; Zhao et al., 2020). The intestinal flora, as an important regulator of intestinal function, has also undergone structural and functional changes in the process of adaptation to the plateau, improving sugar metabolism and fat accumulation. (Backhed et al., 2004; Bäckhed et al., 2007; Krajmalnik-Brown et al., 2012; Tremaroli and Bäckhed, 2012; Qi et al., 2020). However, whether these changes occur in individuals with a fast POL remains unclear. Undoubtedly, enhancing the nutrient absorption efficiency of animals inhabiting high-altitude places may enable positive individuals to maintain a high energy-consuming life rhythm.

The toad-headed lizard *Phrynocephalus vlangalii*, which is widely distributed on the Tibetan Plateau, covers altitudes ranging from 2300–4500 m (Zhao, 1979) and is an ideal organism for ecological studies (Jin et al., 2013; Han et al., 2016). Some studies show that *P. vlangalii* at high altitudes has a slow life history strategy to reduce energy consumption; however, simultaneously, its intestinal tract and flora evolve, which further enhances its energy absorption capacity (Jn and Liu, 2013; Li et al., 2014; Han et al., 2016; Zhang et al., 2018). However, whether there are different personalities in the high-altitude population of *P. vlangalii* and whether the intestinal microorganisms are different between individuals with varying POL remain unknown. Therefore, it is necessary to design this experiment to study whether lizards at high altitude have different personalities and to find out the differences of intestinal microbes between two individuals with different POLs. In this study, the personality and gut microbes of one population of *P. vlangalii* were studied to test the following two hypotheses: (1) the personalities and POL of *P. vlangalii* individuals inhabiting high-altitude places are remarkably distinguishable, and (2) gut microbes may help the host maintain a stable personality through certain energy metabolic pathways and immune mechanisms.

## 2 Materials and methods

### 2.1 Study area and sampling

To avoid the effect of female reproduction and possible seasonal changes in energy distribution, 37 adult male *P. vlangalii* were captured manually from MaDuo (MD) (N 34°36′20.99′′, E 98°7′49.83′′, 4250 m), Qinghai Province, China, in early August 2020. To eliminate differences between individuals caused by age, after measuring each snout vent length, 33 lizards of similar size were selected for this study (**Table S1**). The animal study protocol was reviewed and approved by Lanzhou University’s Institutional Animal Care and Use Committee.

To accurately reflect the microbial flora in the wild, the lizards were disinfected with 75% alcohol and maintained individually in sterile boxes immediately after capture. Boxes were checked every 30 min to attempt feces collection within 30 min after defecation. Feces were kept separately in 1.5 ml sterile polypropylene tubes and frozen immediately at −20°C. The lizards and feces were then taken to the laboratory. The lizards were kept in individual terraria (550 × 395 × 337 mm) with 5 cm of wet sand and a small brick shelter. A photoperiod of 12 h light and 12 h dark was set by fluorescent lamps, and the room temperature was set at 20°C by air conditioner. A 50-W heat cable was hung 10 cm above the sand to create a thermal gradient ranging from 20°C–50°C. Fresh mealworms (*Tenebrio molitor*) and water supplemented with vitamins were supplied daily.

### 2.2 Growth rate

To confirm whether the lizards from high altitudes possessed different types of POL, the growth rate during the experiment was calculated. As the growth rate of *P. vlangalii* lizards is slow after sexual maturity (Zhao et al. 2020), the measurement error of snout vent length variation during the short feeding period could not be ignored. Therefore, the growth rate of the body mass was used. The body weight of each lizard was measured before and after all behavioral trials using an electronic scale (0.01 g), and the growth rate was calculated as the increase in body mass per day.

### 2.3 Behavioral assays

Two energy-intensive behavioral characteristics of each lizard were tested thrice from early August to mid-September 2020 (once every 2 weeks). To control the influence of daily rhythms, the assays were only conducted between 10:00 am and 12:30 am, during the peak activity period of *P. vlangalii* in the field. A hidden camera system was used to record the behavior, and the video was checked by an individual researcher to ensure consistency.

#### 2.3.1 Boldness

Boldness was measured as the time it took for a lizard to leave an unfavorable refuge after a simulated predatory attack following Baxter-Gilbert et al. (2019). A plastic bin (390 W×510 L×320 H mm) with a temperature heterogeneity that was created by a “cold” refuge and a “hot” refuge was used in this test (Riley et al., 2017; Damas-Moreira et al., 2019). The lizard was first introduced into the arena to acclimate for 5 min and then driven to the “cold” refuge by a gloved hand. Finally, we recorded the lizard behavior for 45 min and scored boldness as the latency from when the lizard entered the cold shelter until it emerged. The shorter the time, the higher the braveness. If the lizard did not leave the refuge, the recording time was set at 2700 s. In the subsequent repeatability verification test, gloves of different materials and colors were used to prevent lizards from adapting.

#### 2.3.2 Foraging activity

Foraging activity was measured as the number of successful attacks given prey in a given period of time following a previous study (Michelangeli et al., 2015). The lizards were placed in a test arena with shelter at one end. After 5 min of acclimation, five mealworms of equal size were dropped into the arena by an observer behind a curtain. We then recorded the number of mealworms consumed by the lizard and the number of failed capture attempts made by the lizard—failed capture attempts included prey drops (when a mealworm was caught but then dropped) and any lunge attack that failed to capture a mealworm—over 15 min. In the second repeatable test, crickets were used as prey to prevent the lizards from becoming habituated. Similarly, in the third experiment, we removed fine sand from the bottom of the test arena to prevent the lizards from familiarizing themselves with their foraging environments. The lizards were not fed for 3 days before each trial. The foraging score for each lizard was calculated as follows:


$$Foraging\;score=\frac{\;\left(\text{number of prey eaten}/\text{total number of prey offered}\right)}{\left(\text{total number of capture attempts}/\text{number of successful attempts}\right)}$$

### 2.4 DNA extraction and sequencing

According to the results of the behavioral analysis, the feces of 16 lizards from the Pos and Shy groups (eight in each group, see below for details) were selected for DNA extraction and sequencing. Total genomic DNA was extracted using a fecal DNA extraction kit from Shenggong Company (dp328, Shenggong, Shanghai). Primers 515f and 806r were used to amplify the V3 and V4 regions of the 16S rRNA gene, respectively, in each sample. The PCR system consisted of 4 μL 5 × FastPfu polymerase, 0.2 μ L dNTPs (2.5 mm) BSA, 10 ng DNA template, and 20 μL water. Thermal cycling consisted of an initial denaturation at 98°C for 1 min, followed by 30 cycles of denaturation at 98°C for 10 s, annealing at 50°C for 30 s, and elongation at 72°C for 60 s. The PCR system was stored at 72°C for 7 min after 4 min, and a 2% agarose gel was used to detect the PCR products. The AxyPrep DNA Gel Extraction Kit was used to purify the PCR products for further experiments.

DNA libraries were constructed using a TruSeq^®^ DNA PCR-Free Sample Preparation Kit (Illumina, San Diego, CA, USA) following the manufacturer’s recommendations, and index codes were added. The components of the libraries were then sequenced on an Illumina Nova platform, and paired-end sequencing was performed using sequencing strategy PE250 with a fragment size of read length of 450–550 bp.

### 2.5 Statistical analysis

#### 2.5.1 Behavioral traits analysis

The consistency of boldness and foraging activity of *P*. *vlangalii* was estimated by calculating the adjusted repeatability with 95% confidence intervals for the two behaviors. We used the rpt function from the R package rptR to calculate the repeatability coefficient (ICC) (Nakagawa and Schielzeth, 2010). To assess the significance of Radj, we visually examined whether the 95% CIs for each estimate included a zero. We used the plot function in the R package to visualize the correlation relationship between behaviors, the least square method to fit the correlation regression model, and the pt function in the R package to calculate the *P*-value to determine the presence of a linear relationship.

The boldness score was used to divide the lizards into positive and shy lizards. We used SPSS 2.0 to calculate the quartile of the 33 samples (ranked from strongest to weakest in terms of boldness), eight of the “smaller quartile” (Q1) as the positive group (Pos), eight of the “larger fourth quartile” (Q3) as the shy group (Shy), and 17 of the “median” (Q2) as the Mid group. The differences in growth rates between the Pos and Shy groups were estimated using a *t*-test.

#### 2.5.2 Gut microbe analysis

The abundance of microbial taxa was expressed as a percentage of the total 16S rRNA gene sequences, and the differences between groups were compared. To evaluate the complexity of the intestinal microbiota, observed OTUs, alpha diversity (Shannon), richness (Chao), Good’s coverage, and beta diversity were calculated using QIIME (v1.9.0) based on both weighted and unweighted UniFrac metrics and displayed with R software (v2.15.3). To identify differences in microbial communities between the two groups, analysis of similarities (Clarke, 1993) was performed based on the Bray–Curtis dissimilarity distance matrices using the vegan package in the R software. The Bray–Curtis distance between the microbial communities of all individuals from the positive or shy group was calculated using the logarithm of the dissimilarity coefficient. The *t*-test was then used to calculate the significance of β diversity. Principal component analysis was performed at the phylum and genus levels, and the results were visualized using the STAMP version 2.1.3 program (Parks et al., 2014). LEfSe analysis was conducted to compare the taxonomic abundance of the intestinal microbiota composition among the different personalities. The Bray–Curtis distance was represented using an unweighted pair group method with arithmetic mean clustering tree. To predict each metagenome based on the 16S rRNA amplicon sequences, prediction of microbial function was performed using PICRUST version 1.0.0 (http://picrust.github.io/picrust/) programs. The OTU data were refined and normalized based on the predicted 16S rRNA gene copy number, and the precalculated Kyoto Encyclopedia of Genes and Genome (KEGG) orthologs database (https://www.genome.jp/kegg/) was used to predict the metagenomics. The predicted metagenomes were collapsed into a specified level in a hierarchy using KEGG pathway metadata and analyzed using the STAMP program. The relative abundance of the KEGG pathway was compared between the two groups using two-sided Welch’s *t*-test (Parks et al., 2014).

## 3 Results

### 3.1 Behavioral traits correlation

Considerable variability in boldness test hiding time was noticed over the entire data set with the boldest lizard hiding for 31 s and the shyest hiding for 8100 s. There was a highly significant individual lizard effect in the boldness test [**Table S1**; ICC = 0.848, *p*<.001]. In the foraging activity test, the sum of the most active lizard’s foraging score was 2.83, and that of the shyest was 0.70. There was also a significant difference in the foraging activity test results [**Table S1**; ICC = 0.424, *p*<.001]. Body size of *P*. *vlangalii* had no significant effect on both behaviors as SVL was not significantly associated with foraging activity (*p* = .481, *r* = –0.127) and boldness (*p* = .549, *r* = 0.108), respectively.

The increase in body weight in the Pos group was significantly greater than that in the Shy group [**Table S2**; *t*-test, *p* = .021].

**Figure 1:**
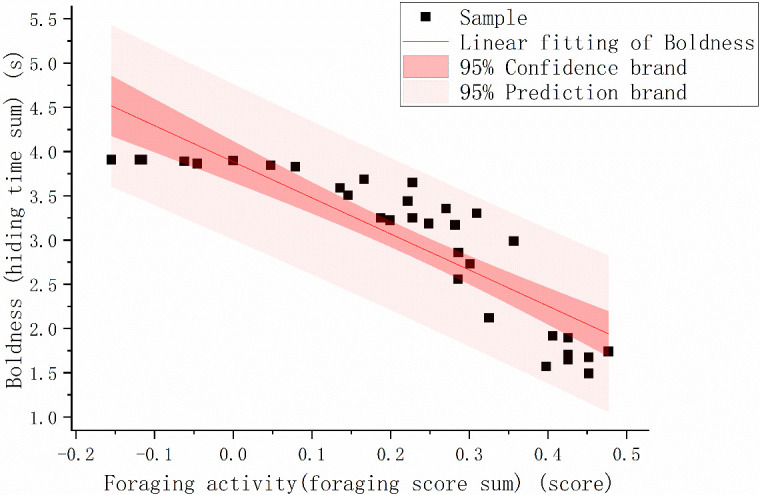
The foraging activity (foraging score sum) and boldness (hiding time sum) scatter map of *Phrynocephalus vlangalii*. We plotted fitted lines predicted from our linear models with 95% confidence intervals. To better visualize the data, we take the logarithm of 10 for the two sets of data.

Regression analysis showed that individuals that exhibited more foraging behavior were bolder [**Figure 1**; *p*<.001, *r* = –0.876].

### 3.2 Microbial community composition

**Figure 2:**
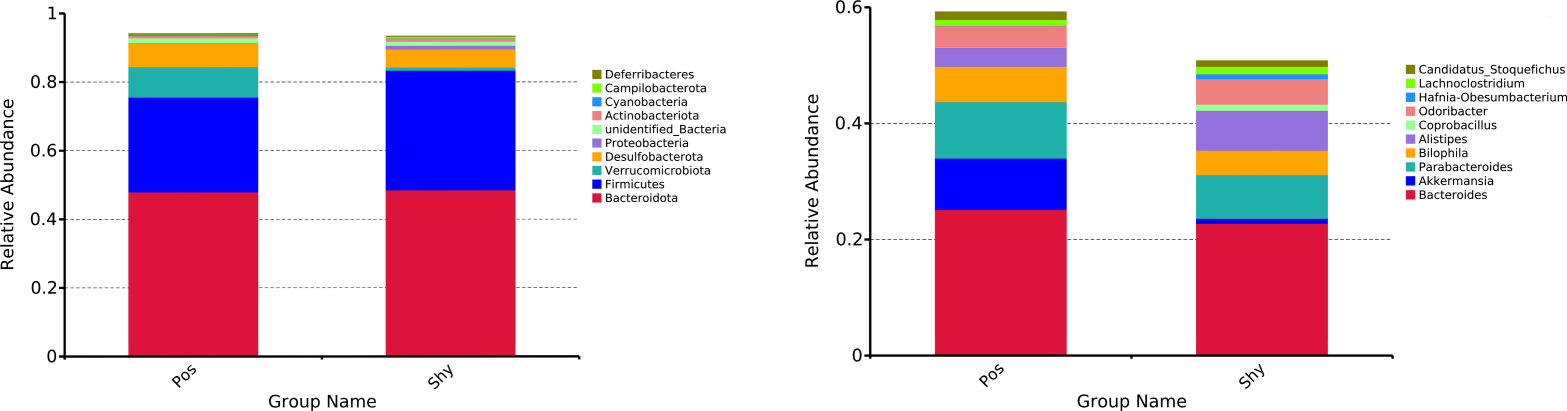
Relative abundances of microbial phylum of *P. vlangalii* represented as bar plots at the phylum level (left) and genus level (right). Only the top 10 phylum and top 10 genera are shown in the histogram, and the other taxa are combined. Pos and Shy indicate positive personality group and shy personality group.

A total of 1,264,002 high-quality (>Q30) reads were filtered from 1,473,978 raw reads obtained from 16 intestinal content samples. At a 97% similarity level, 9005 OTUs classified into 18 phyla were obtained (**Figure S1**), and the rank abundance curves of all samples gradually stabilized, indicating a uniform species distribution (**Figure S2**). At the phylum level, Bacteroidetes, Firmicutes, and Verrucomicrobiota were the dominant phyla in the Pos. The dominant phyla in the Shy group were Bacteroidetes, Firmicutes, and Desulfobacterota. At the genus level, the dominant genera in the Pos group were *Bacteroides*, *Akkermansia*, and *Parabacteroides* species. However, the dominant genera in the Shy group were *Bacteroides*, *Parabacteroides*, and *Alistipes* (**Figure 2**).

### 3.3 Microbial community structure

**Figure 3:**
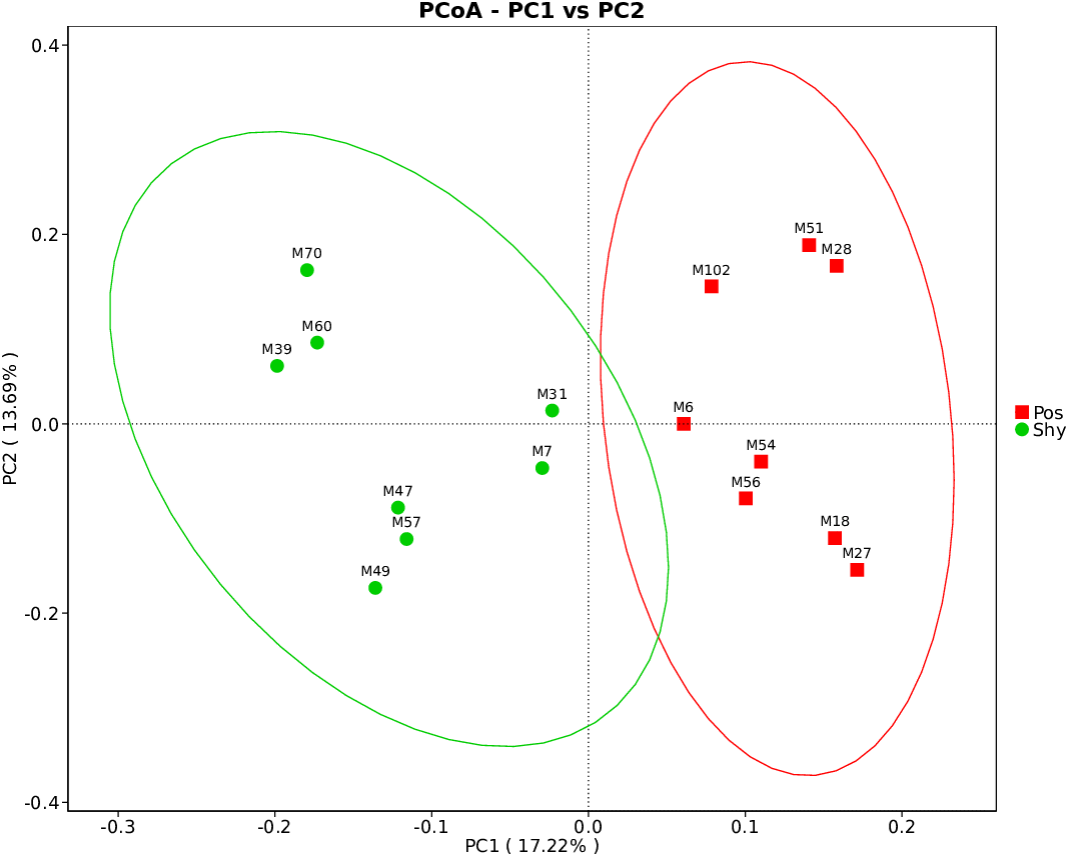
PCoA of Bray–Curtis distance metric of intestinal microbiota of the lizard based on different personalities. Pos: Positive *P. vlangalii*. Shy: Shy *P. vlangalii*.

Alpha diversity of intestinal microbiota was assessed using the observed species, Shannon, Simpson, ACE, and Chao1 indices. There was no significant difference in α diversity between the Pos and Shy groups with different personalities (*p* = .6454, **Supplementary Figure 1**; **Table S4**). However, there was a significant difference in the β-diversity between the two groups (*p* = .047, **Supplementary Figure 2**). We performed Bray–Curtis principal coordinate analysis (PCoA) and found an obvious division of different personalities. Pco1 and Pco2 accounted for 17.22% and 13.69% of the total variation, respectively (**Figure 3**).

**Figure 4:**
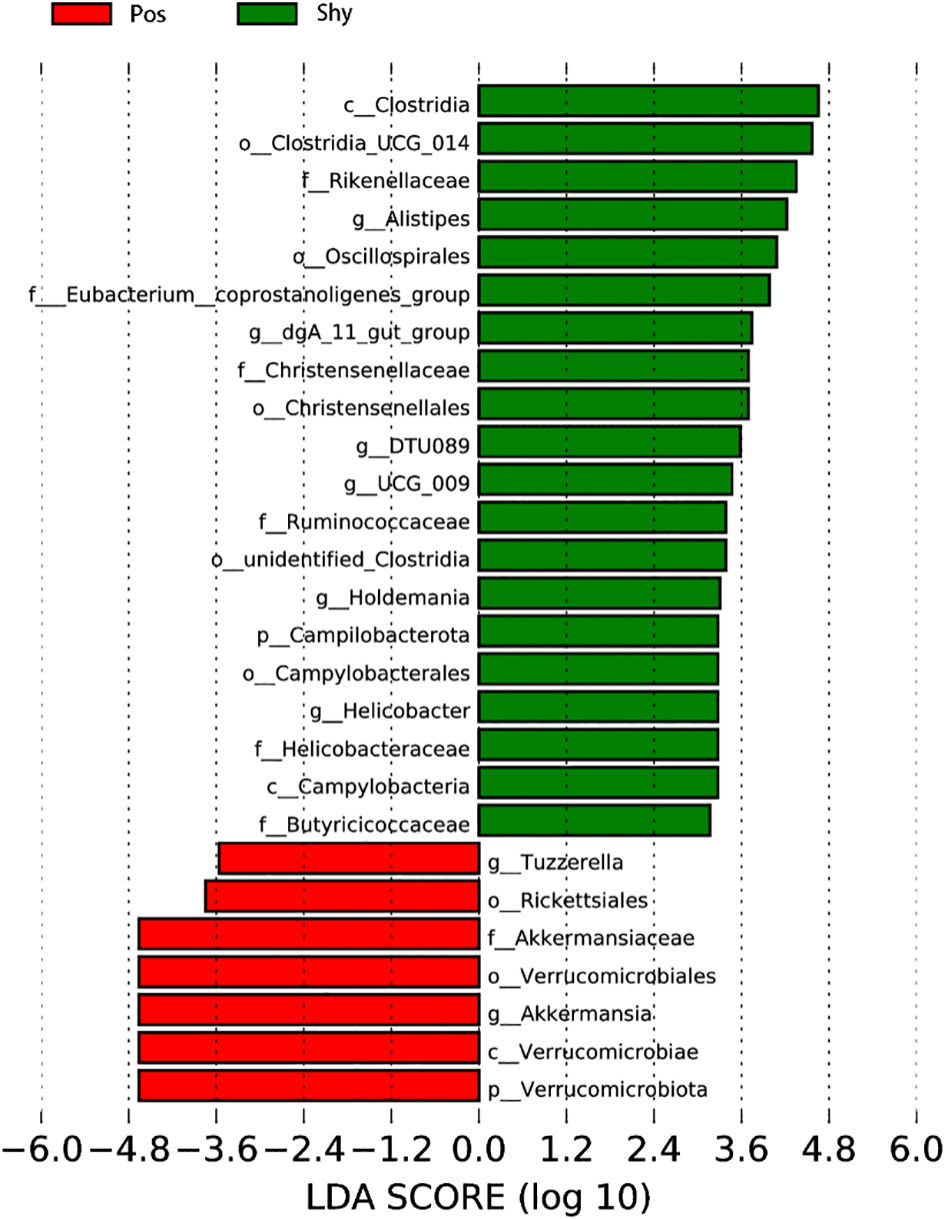
Using LEfSe software (linear discriminant analysis [LDA]), the differential abundance of several taxa in the gut microbiota of the individual *Phrynocephalus vlangalii* lizard was analyzed. Taxa enriched in Pos group are indicated with a positive LDA score (red), whereas taxa enriched in Shy group have a negative score (green).

The LefSe analysis revealed a greater proportion of the family Rikenellaceae and genus *Clostridia* in shy lizards (Shy) than in positive lizards (Pos). In contrast, the proportions of the phylum Verrucomicrobiota, order Verrucomicrobiae, and family Akkermansiaceae in intestinal microbiota of Pos were greater than Shy (**Figure 4**).

### 3.4 Differential KEGG pathways among the gut microbiota

**Figure 5:**
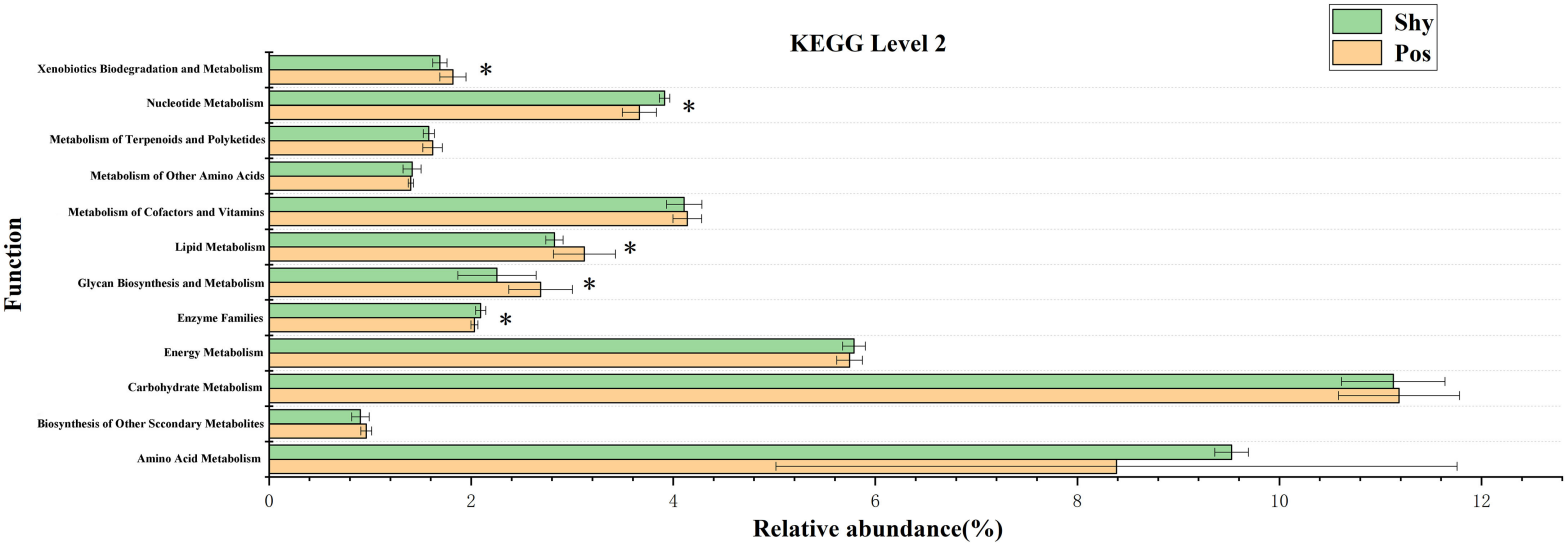
Differential abundance of several taxa in gut microbiota of Pos vs. Shy. The differences in the various level compositions were tested using a two-sided Welch’s *t*-test, and *P*<.05 was considered as significant (mark “*“ symbol). Pos: Positive *P. vlangalii*. Shy: Shy *P. vlangalii*.

After classifying all KEGG orthology (KO) into second-level functions, a large number of differences were found between the two groups in the KEGG pathway. Here, we screened for different metabolism-related KEGG pathways (**Figure 5**). Nucleotide metabolism and enzyme families were significantly higher in the microbiota of the shy personality group. Biodegradation and metabolism, lipid metabolism, and glycan biosynthesis and metabolism were significantly higher in the microbiota of the positive personality group.

## 4 Discussion

Our research is devoted to linking the energy-intensive personalities of animals with their intestinal microflora under conditions of energy limitation in high-altitude areas. By evaluating the repeatability of bravery, we verified that there is indeed an energy-intensive personality in *P. vlangalii*, even under the condition of energy limitation. The significant correlation between boldness and growth rate further proved that these braver individuals possess fast POL (**Figure 1**). The correlation between boldness and foraging activity and the difference in gut microbes between positive and shy individuals verified our previous hypothesis that intestinal microbes may help the host maintain its energy-intensive personality.

Diet is one of the most important factors affecting intestinal microorganisms (Song et al., 2013; David et al., 2014); the more diverse the diet, the more diverse the gut microbiota (Laparra and Sanz, 2010). According to the increased intake model, positive individuals are more active during forage than are Shy individuals (Biro and Stamps, 2008); therefore, they may carry more diverse microbes. In our study, although *P. vlangalii* from the Pos group showed stronger foraging behavior than those from the Shy group, the α diversity of the gut microbiota did not increase significantly (**Supplementary Figure 1**; **Table S4**). This may be related to the limited number of food species at high altitudes because the diversity and abundance of insects decreases with increasing altitude (Joshi et al., 2008). However, our study found significant differences in the β diversity of gut microbiota between the Pos and Shy groups in *P. vlangalii* (**Table S5**), which may be related to the difference in foraging preference or habitat between the two groups because the beta diversity of gut microbiota of an individual was positively correlated with that of its diet (Li et al., 2016). In general, braver individuals are more likely to forage further away on their own (Holbrook and Schmitt, 1992). Shy individuals, whose gut microbes contain more Rickettsiales (**Figure 4**), an obligate intracellular parasite that can be transmitted by parasites in animals (Thomas et al., 2016), also indicated that they tend to forage in small groups near nests. The differences in the intestinal microbial structure between the two groups of *P. vlangalii* were further confirmed by PCoA (**Figure 3**) and LefSe analysis (**Figure 4**).

Shy individuals of *P. vlangalii*, which possess a slow POL with a low growth rate, long longevity, and high energy storage, have a series of microbes that are related to these traits. First, Shy *P. vlangalii* carry a higher abundance of Clostridia and Rikenellaceae, which can help maintain metabolic stability in aged individuals (**Figure 4**). The high abundance of Clostridia can constrain lipid metabolism to prevent age-associated metabolic syndrome (Petersen et al., 2019), which helps reduce metabolic disorders, such as hypertension, hyperglycemia, and visceral fat excess caused by aging (Ford et al., 2002). Similarly, high abundance of Rikenellaceae (**Figure 4**) was associated with reduced visceral adipose tissue and a healthier metabolic profile in aged individuals (Tavella et al., 2021). Second, to store energy for a long time, Shy individuals possess more microbes, such as Clostridia_UCG-014 and Ruminococcaceae (**Figure 4**), which can increase fasting blood glucose (FBG) and insulin tolerance. High abundance of Clostridia_UCG-014 is shown to correlate with FBG (Zhao et al., 2021) and can facilitate energy transfer after forage of Shy individuals. The high FBG level of Shy individuals is an insulin-tolerant constitution. Species of Ruminococcaceae are strongly correlated with the level of blood urea nitrogen (Shao et al., 2019), which can suppress insulin secretion and sensitivity (Xie et al., 2018). Finally, in Shy individuals, the relative abundance of Oscillospirales (**Figure 4**), which is of great significance to the ecological balance and diversity of intestinal microbes (Xie et al., 2021), was also higher and may contribute to the niche stability of Shy individuals in their long-life POL.

The Pos individuals of *P. vlangalii* that grow fast, are more active, and live short lives also formed a differentiated intestinal microbial structure that was compatible with their high energy consumption behavior (**Figure 4**). For example, the abundance of Akkermansia muciniphila, which is positively correlated with a decrease in FBG and total cholesterol levels after fasting (Özkul et al., 2019), is shown to be negatively correlated with body mass. Akkermansia muciniphila is important for improving host metabolic functions and may be helpful in maintaining active POL in Pos lizards at high altitudes. In addition, the abundance of Verrucomicrobia, which contributes to the control of energy homeostasis and glucose metabolism (Cani et al., 2014), was also higher in Pos lizards. That is, Pos lizards choose higher sugar metabolism, lipid consumption, and storage of less lipids to maintain their fast POL.

KO analysis also showed specific energy metabolism pathways and enzyme systems in accordance with different types of POL in *P. vlangalii* (**Figure 4**). In accordance with the high energy consumption and exploratory nature of Pos lizards, xenobiotic biodegradation and metabolism, lipid metabolism, and glycan biosynthesis and metabolism in the microbiota were significantly higher than those in Shy lizards. First, due to the high abundance of Pos individuals, they are exposed to more xenobiotics, resulting in high biodegradation and metabolism of xenobiotics. Studies show that lipid metabolism is strengthened in *P. vlangalii* at high altitudes (Tang, 2013). A stronger fatty acid metabolism can provide more energy for Pos, which also shows that Pos individuals are not eager to store energy. Finally, to achieve higher metabolism, the intestinal microbes of Pos lizards have more influence on the biosynthesis and metabolism of glycogen, which is often closely related to the metabolism of organisms (Roach et al., 2012). Of note, the enzyme family, which plays an important role in cell metabolism, organism homeostasis, and health (Zhu et al., 2019), is greater in Shy than in Pos, indicating that Shy, which adopts slow POL, chooses to invest more energy and resources in maintenance.

## 5 Conclusion

In this study, boldness and foraging behavior of *P. vlangalii* from Maduo (4250 m a.s.l.), a typical slow POL population, were screened repeatedly. Both behaviors of *P*. *vlangalii* repeated significantly and correlated with each other. Based on the boldness, lizards were divided into positive (Pos) and shy (Shy) groups. No significant difference in α diversity was found, but a significant difference exists in the β diversity of gut microbes between two groups. PCoA confirmed that the gut microbes of the two groups were clearly separated. The LefSe analysis indicated that the Shy group carried a greater proportion of Rikenellaceae and *Clostridia*, whereas the Pos group carried higher proportions of Verrucomicrobiota, Verrucomicrobiae, and Akkermansiaceae. KEGG pathway analysis found that biodegradation and metabolism, lipid metabolism, and glycan biosynthesis and metabolism showed higher levels in the Pos group; however, nucleotide metabolism and enzyme families were significantly higher in the Shy group. Therefore, the Pos lizards exhibited intestinal microflora that is more beneficial to its lipid and glucose metabolism, thus meeting its high metabolic energy demand, whereas the intestinal microflora of Shy lizards is beneficial to maintain high fasting blood glucose, long longevity, more stable metabolism, and help in maintaining its own slow POL. Overall, we confirm that there is a significant relationship between an individual’s POL or personality and its intestinal microorganisms in *P*. *vlangalii* and that gut microbes play a beneficial role in maintaining the energy-intensive personality in high altitude regions.

## Data availability statement

The data presented in the study are deposited in the NCBI database repository, accession number PRJNA850661.

## Ethics statement

The animal study was reviewed and approved by Lanzhou University’s Institutional Animal Care and Use Committee, Lanzhou University.

## Author contributions

ZB: Manuscript editing and writing; WZ: Manuscript revision, Experimental design; CY: Behavioral experiment, Data analysis and drawing; CN: Experimental operation, Data analysis and drawing; YQ: Sample collection, Experimental material acquisition; YZ: Sample collection, Experimental material acquisition; SC: Sample collection, Experimental material acquisition. All authors contributed to the article and approved the submitted version.

## Conflict of interest

The authors declare that the research was conducted in the absence of any commercial or financial relationships that could be construed as a potential conflict of interest.

## Publisher’s note

All claims expressed in this article are solely those of the authors and do not necessarily represent those of their affiliated organizations, or those of the publisher, the editors and the reviewers. Any product that may be evaluated in this article, or claim that may be made by its manufacturer, is not guaranteed or endorsed by the publisher.
