# Supplementary figures and images for: Beneficial role of gut microbes in maintenance of pace-of-life traits in Phrynocephalus vlangalii
Source: Front Microbiomes. 2022 Nov 21;1:962761. doi: 10.3389/frmbi.2022.962761 (PMC12993462; doi:10.3389/frmbi.2022.962761)

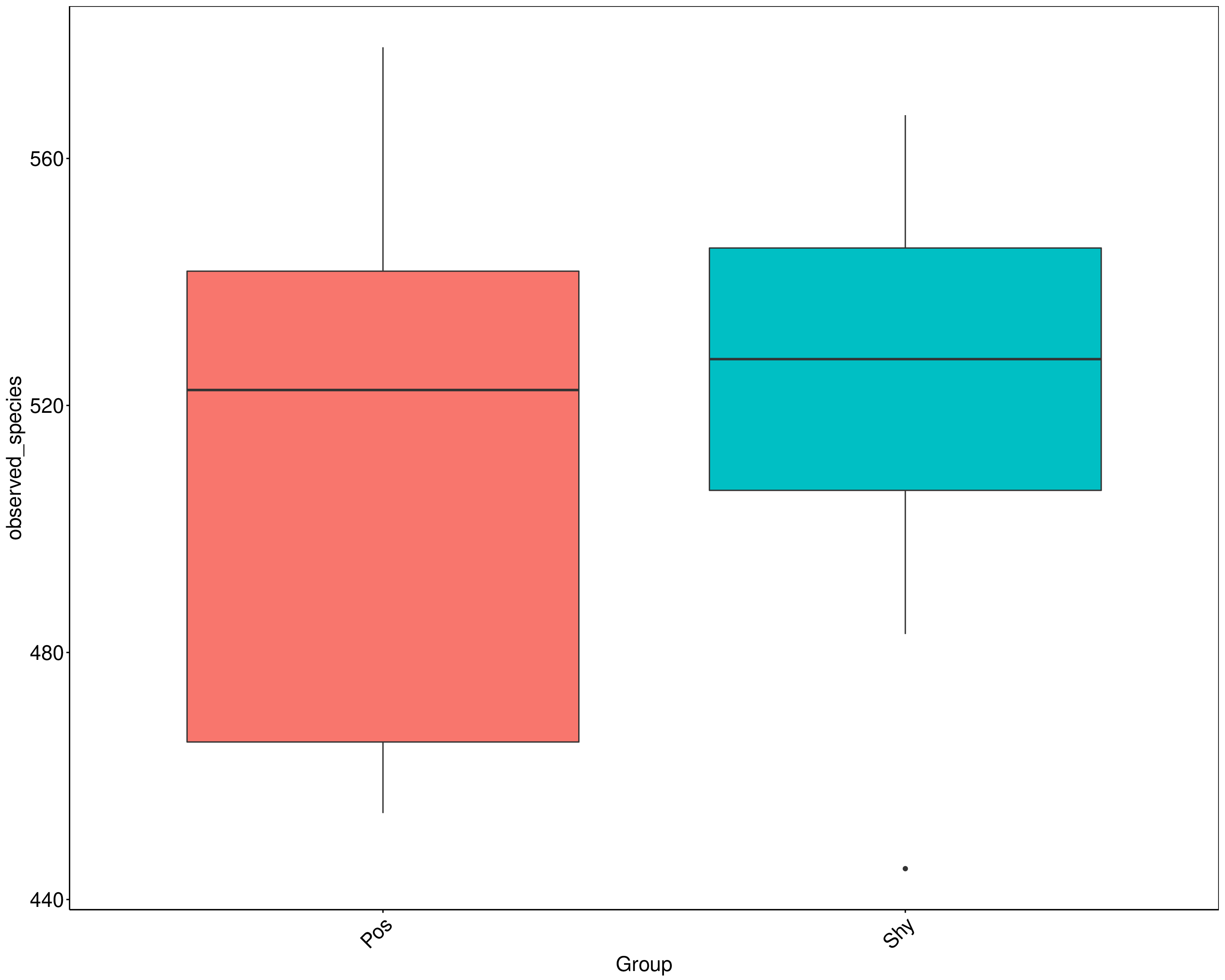

Supplement: Supplementary file 1 [file Image_1.tiff]

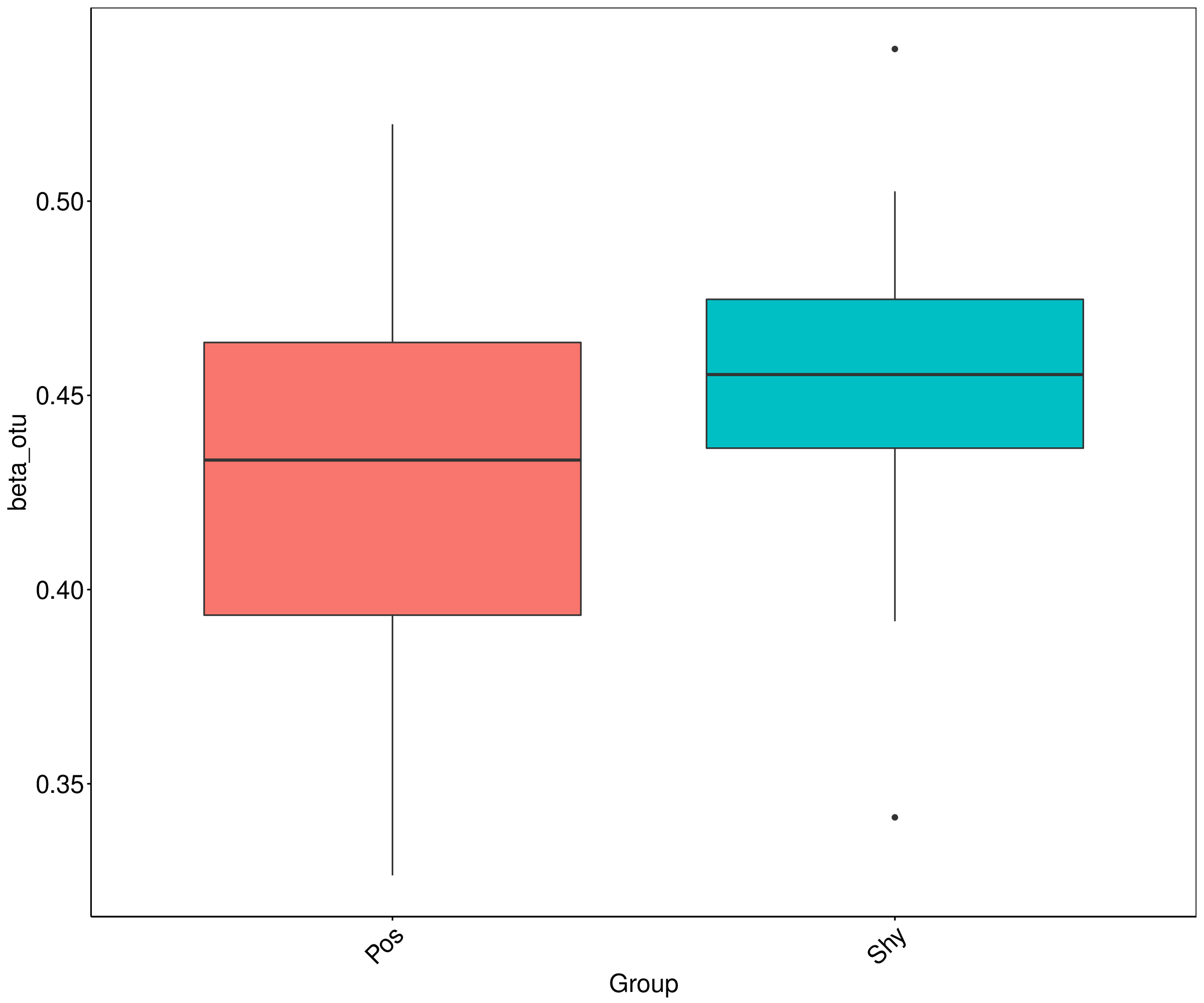

Supplement: Supplementary file 2 [file Image_2.tiff]
