# Supplementary material for: Beneficial role of gut microbes in maintenance of pace-of-life traits in Phrynocephalus vlangalii
Source: Front Microbiomes. 2022 Nov 21;1:962761. doi: 10.3389/frmbi.2022.962761 (PMC12993462; doi:10.3389/frmbi.2022.962761)
Supplement: Supplementary file 3 [file Table_1.docx]

**Table S1**

The results of the two personality tests (the sum of the three replicates) and SVL.

| **Group** | **Sample**  **number** | **Boldness test hiding time sum（s）** | **Foraging score sum(score)** | **SVL(mm)** |
| --- | --- | --- | --- | --- |
| Pos(C1) | M51 | 31 | 2.83 | 55.38 |
|  | M28 | 37 | 2.50 | 54.59 |
|  | M102 | 44 | 2.67 | 54.30 |
|  | M18 | 47 | 2.83 | 57.19 |
|  | M56 | 51 | 2.67 | 53.16 |
|  | M6 | 55 | 3.00 | 56.08 |
|  | M54 | 78 | 2.67 | 58.84 |
|  | M27 | 82 | 2.55 | 53.50 |
| Mid(C2) | M8 | 132 | 2.11 | 60.67 |
|  | M12 | 362 | 1.93 | 58.79 |
|  | M15 | 536 | 2.00 | 55.69 |
|  | M21 | 721 | 1.93 | 60.13 |
|  | M23 | 974 | 2.27 | 61.20 |
|  | M61 | 1482 | 1.91 | 53.71 |
|  | M50 | 1532 | 1.77 | 54.88 |
|  | M55 | 1671 | 1.58 | 54.24 |
|  | M58 | 1779 | 1.69 | 59.25 |
|  | M69 | 1783 | 1.54 | 55.39 |
|  | M67 | 2003 | 2.04 | 58.86 |
|  | M59 | 2259 | 1.87 | 58.91 |
|  | M63 | 2762 | 1.67 | 56.83 |
|  | M66 | 3198 | 1.40 | 56.79 |
|  | M77 | 3872 | 1.37 | 55.10 |
|  | M101 | 4476 | 1.69 | 59.99 |
|  | M103 | 4872 | 1.47 | 55.24 |
| Shy(C3) | M31 | 6721 | 1.20 | 56.04 |
|  | M7 | 6982 | 1.12 | 56.25 |
|  | M49 | 7288 | 0.90 | 57.85 |
|  | M47 | 7767 | 0.87 | 57.12 |
|  | M57 | 7932 | 1.00 | 59.00 |
|  | M70 | 8100 | 0.76 | 54.04 |
|  | M39 | 8100 | 0.70 | 59.23 |
|  | M60 | 8100 | 0.77 | 57.52 |
