# Supplementary material for: Beneficial role of gut microbes in maintenance of pace-of-life traits in Phrynocephalus vlangalii
Source: Front Microbiomes. 2022 Nov 21;1:962761. doi: 10.3389/frmbi.2022.962761 (PMC12993462; doi:10.3389/frmbi.2022.962761)
Supplement: Supplementary file 4 [file Table_2.docx]

**Table S2**

The weight of the two groups after capture and the weight of the lizards after all the behavioral experiments.

|  | **The initial**  **weight(g)** | **Weight after**  **feeding(g)** | **Average rate of**  **change** |
| --- | --- | --- | --- |
| Pos | 7.23±0.81 | 7.761±0.56 | 8.00% |
| Shy | 8.35±0.85 | 7.43±1.31 | -11.32% |
| *P* value |  |  | 0.021 |
