# Supplementary material for: Beneficial role of gut microbes in maintenance of pace-of-life traits in Phrynocephalus vlangalii
Source: Front Microbiomes. 2022 Nov 21;1:962761. doi: 10.3389/frmbi.2022.962761 (PMC12993462; doi:10.3389/frmbi.2022.962761)
Supplement: Supplementary file 5 [file Table_4.docx]

**Table S4**

Data on α diversity of intestinal microbiota in *P. vlangalii*

| **Population** | **Shannon** | **Simpson** | **Chao1** | **ACE** | **Goods_coverage** | **PD_whole_tree** |
| --- | --- | --- | --- | --- | --- | --- |
| Pos | 6.034±0.243 | 0.956±0.013 | 609.9±61.6 | 617.6±56.72 | 0.9973±0.0004 | 35.9±2.34 |
| Shy | 6.263±0.349 | 0.961±0.023 | 636.4±56.6 | 636.0±50.70 | 0.9970±0.0007 | 38.2±3.75 |

All indices were calculated at the 97% similarity level. The number in the parentheses represents the sample size. Pos = positive group, Shy = shy group.
